# Causal analysis of radiotherapy safety incidents based on a hybrid model of HFACS and Bayesian network

**DOI:** 10.3389/fpubh.2024.1351367

**Published:** 2024-05-30

**Authors:** Haiping He, Xudong Peng, Dashuang Luo, Weige Wei, Jing Li, Qiang Wang, Qing Xiao, Guangjun Li, Sen Bai

**Affiliations:** ^1^Department of Radiation Oncology, Cancer Center, West China Hospital, Sichuan University, Chengdu, China; ^2^Department of Radiotherapy Physics & Technology, West China Hospital, Sichuan University, Chengdu, China

**Keywords:** human factors analysis and classification system, Bayesian network, human factors, radiotherapy incidents, patient safety

## Abstract

**Objective:**

This research investigates the role of human factors of all hierarchical levels in radiotherapy safety incidents and examines their interconnections.

**Methods:**

Utilizing the human factor analysis and classification system (HFACS) and Bayesian network (BN) methodologies, we created a BN-HFACS model to comprehensively analyze human factors, integrating the hierarchical structure. We examined 81 radiotherapy incidents from the radiation oncology incident learning system (RO-ILS), conducting a qualitative analysis using HFACS. Subsequently, parametric learning was applied to the derived data, and the prior probabilities of human factors were calculated at each BN-HFACS model level. Finally, a sensitivity analysis was conducted to identify the human factors with the greatest influence on unsafe acts.

**Results:**

The majority of safety incidents reported on RO-ILS were traced back to the treatment planning phase, with skill errors and habitual violations being the primary unsafe acts causing these incidents. The sensitivity analysis highlighted that the condition of the operators, personnel factors, and environmental factors significantly influenced the occurrence of incidents. Additionally, it underscored the importance of organizational climate and organizational process in triggering unsafe acts.

**Conclusion:**

Our findings suggest a strong association between upper-level human factors and unsafe acts among radiotherapy incidents in RO-ILS. To enhance radiation therapy safety and reduce incidents, interventions targeting these key factors are recommended.

## 1 Introduction

Radiotherapy, recognized as one of the safest practices in modern medicine, boasts a remarkably low incidence of procedural errors, approximately 0.2% per fraction delivered (1–3). Nevertheless, the complex nature of radiotherapy, involving intricate interplay and transitions among individuals, leaves room for accidents and radiation errors. These risks threaten the health and safety of both patients and medical staff. The International Atomic Energy Agency categorizes such radiotherapy errors and accidents as “incidents” (4). Incident learning systems, developed internationally, facilitate the reporting of radiotherapy incidents, fostering improvements in radiotherapy quality and patient safety through lessons derived from these incidents.

The concept of incident learning, tracing its origins back to the aviation industry, indicates that 70%−80% of aviation incidents are human factor-related. Intriguingly, a similar trend is seen in radiotherapy safety incidents, with 90% attributed to human factors (5). The human factors analysis and classification system (HFACS) provides a useful framework for examining such incidents. Based on systems theory, HFACS considers unsafe behavior as an initiating point for retrospective incident cause analysis. This approach aids in identifying root causes of incidents and helps in directing safety countermeasures to appropriate levels. HFACS, initially designed for identifying and analyzing aviation accidents (6), has since demonstrated its utility in other areas, including maritime incidents (7), medical errors (8, 9), and transportation mishaps (10, 11), due to its efficacy.

The application of HFACS to radiotherapy is a more recent development. Mosaly et al. (12) compared experts and novices (resident physicians, 2–4 postgraduate years) on 30 radiotherapy incidents after approximately 1 h of training, the results showed that no significant differences were found between novices and experts in the HFACS main heading levels, although the agreement was poorer at the sub-levels. Judy et al. (13), on the other hand, evaluated the consistency of qualitative analytical results amongst radiation oncology professionals, with participants showing 85% and 73% agreement on HFACS main levels and sub-levels in eight incidents. These studies showcased the consistent and reliable implementation of HFACS across various users. In 2009, HFACS model was used in clinical radiotherapy incidents analysis, the study retrospectively analyzed 34 incidents in their radiotherapy center, establishing that recurrent radiation incidents were primarily linked to inattention and mental fatigue (14). Another study (15) expanded beyond assessing the frequency of human factors in 141 incidents, they elucidated the relationship between human factors and different types of radiotherapy errors, finding that skill-based errors can lead to radiotherapy treatment planning errors and decision errors are often associated with quality control errors. This work provides a cornerstone for developing safety precautions for all stages of the radiotherapy process.

Notwithstanding, the application of HFACS in radiotherapy remains relatively nascent and is generally confined to qualitative analysis or basic frequency analysis (5, 14, 16). There is still a lack of integration of HFACS with quantitative analysis methods, and the correlation of human factors in the different levels of the HFACS model remains unclear, which limits the application of HFACS in radiotherapy clinically. To enhance the ability of HFACS to assess human factors in detail during incident investigations, many studies have combined quantitative analysis with the HFACS framework. For example, the HFACS framework has been combined with Bayesian network (BN) (17), fuzzy cognitive mapping (18), analytical network process (19), structural equation model (20), and others. Remarkably, BN is considered to be the most effective method for analyzing dependencies between factors in uncertain research environments and is widely used in the field of safety (17), with previous studies demonstrating the usefulness of integrating BN with HFACS for analyzing accidents and system failures to identify effective control measures (21, 22), which unfortunately has not yet been used in the field of radiotherapy. Therefore, this study seeks to integrate HFACS with BN, explore relationships among contributing human factors in radiotherapy incidents, and to evaluate the impact of upper-level human factors on lower-level human factors. These upper-level factors are commonly perceived as latent factors leading to incident occurrences and are often overlooked in traditional incident analysis. We believe that investigating latent security breaches is key to developing targeted security precautions for radiotherapy procedures.

## 2 Methods

### 2.1 HFACS framework

**Figure 1:**
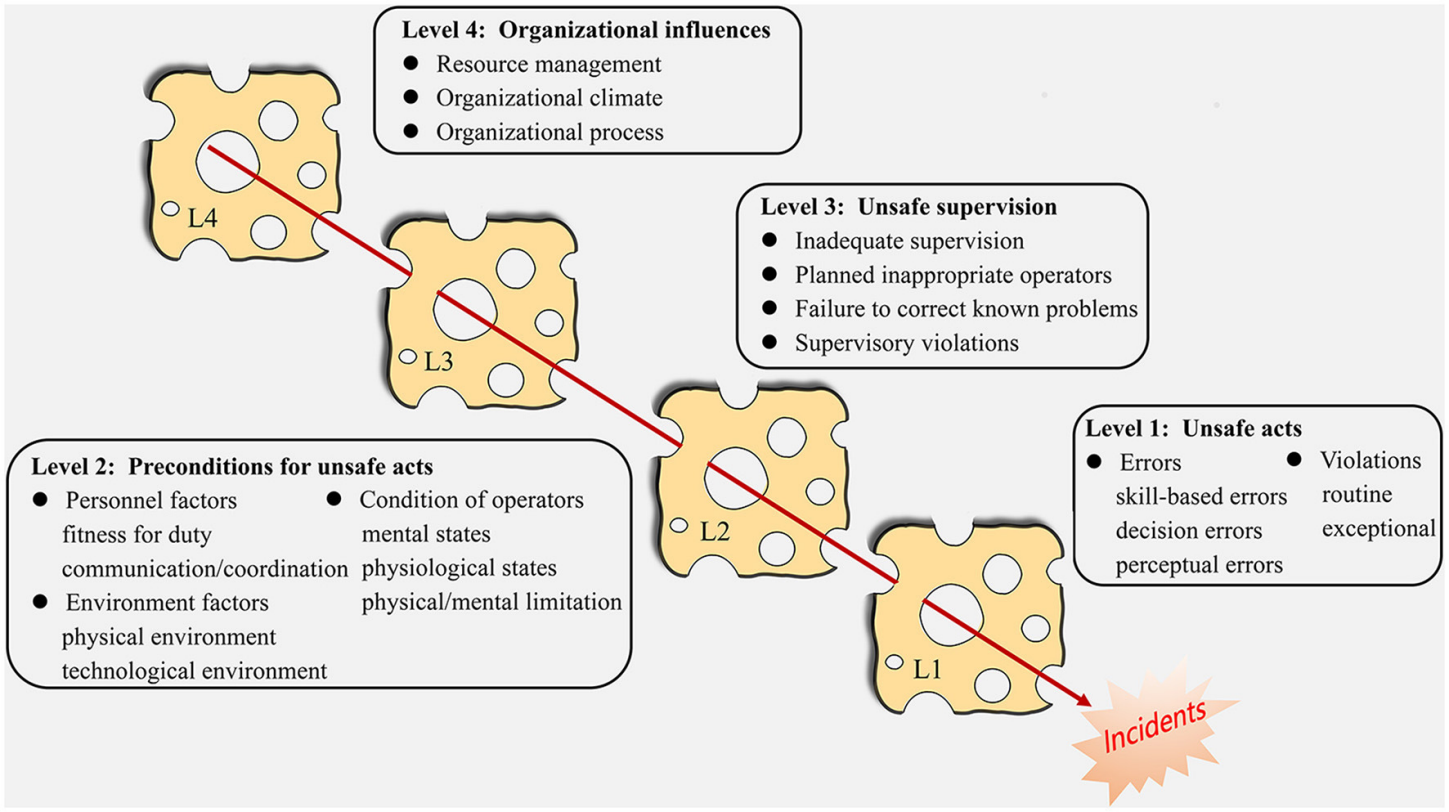
Human factors analysis and classification system framework.

Inspired by Reason's “Swiss Cheese” model, the HFACS model, developed by Shappell and Wiegmann, categorizes accident causes into four levels, likening failures to “vulnerabilities” within each level (6). The HFACS distinguishes between overt and latent human factors based on Reason's model. The overt factors align with the first level of Reason's model—unsafe acts, the active elements directly resulting in accidents. The latent factors correspond to the remaining three levels: preconditions for unsafe acts, unsafe supervision, and organizational influence, respectively. Figure 1 presents a schematic representation of the HFACS, outlining the sub-levels and elucidating the hierarchical relationships among them.

### 2.2 Data source

This study utilized radiotherapy safety incidents sourced from the RO-ILS. The RO-ILS, developed by the American Society for Radiation Oncology (ASTRO) and the American Association of Medical Physicists (AAPM), currently houses a substantial collection of radiotherapy incidents (23). Since 2014, RO-ILS has conducted regular radiotherapy incident sharing with the aim of facilitating knowledge sharing and learning experiences, enabling practitioners in the field of radiotherapy to learn from the incidents of others to improve the quality and safety of patient care. Our study used all the radiotherapy incidents publicly posted by RO-ILS. Indeed, the formulation of error reporting in the RO-ILS system bears a striking resemblance to the HFACS approach (12). Given the absence of brachytherapy at our center, we excluded such incidents to prevent any potential analytical inaccuracies stemming from unfamiliarity. Moreover, incidents lacking discrete cause descriptions (e.g., detailing who was involved and the specific process leading to the incident) were also excluded. This selection process yielded a total of 81 incidents for our study.

### 2.3 Qualitative analysis

In the qualitative analysis process, two experienced physicists, both trained with systematic training in HFACS-related theory and practice, collaboratively evaluated the radiotherapy incidents. Employing a layered indexing approach, the physicists assessed the incident reports to determine the presence or absence of all sub-factors in each of the HFACS model. Notably, the qualitative analysis was a collective effort, with both physicists actively engaging in discussions to achieve consensus on the classification of factors.

In instances where divergent classifications emerged, we engaged additional physicists, all possessing expertise in safety quality assurance of radiotherapy clinical process to participate in collaborative discussions. Through these collaborative efforts, disagreements were thoroughly examined to achieve consensus. By leveraging the collective expertise of a team, we aimed to fortify the accuracy and credibility of our findings, ultimately mitigating concerns related to potential biases or oversights in the qualitative analysis process.

### 2.4 Bayesian network

BN is a model used for characterizing variable dependencies, facilitating quantitative analysis of causal relationships between incident factors and aiding intervention selection (24). The composition of the BN is divided into a qualitative part and a quantitative part. At the qualitative level, a directed acyclic graph (DAG) is used to represent the dependence and independence between two sets of variables; at the quantitative level, a conditional probability table (CPT) is used to describe the dependence between variables (18). A BN is mathematically represented as N = < G, P>, where G signifies a directed acyclic graph DAG. The DAG can be further described as G = < V, E>, with V = {*x*_1_, *x*_2_, …, *x*_*n*_}, representing the set of nodes in the network and E denoting the set of directed edges. The nodes symbolize random variables, while the directed edges depict the relationships between these nodes. P represents the CPT, indicating the strength of logical relationships between nodes. Utilizing the logical node relationships and Bayes' formula, the BN enables probabilistic inferences for uncertainty. The joint probability for any random variable *x* in the BN can be expressed as (Equation 1):


(1) $$\begin{array}{l} P\left(x_{1},x_{2},\ldots ,x_{n}\right)=\overset{n}{\underset{i=1}{\prod }}P\;\left(x_{i}|Pa\;\left(x_{i}\right)\right) \end{array}$$

*Pa* (*x*_*i*_) symbolizes the parent node of *x*_*i*_. *P* (*x*_*i*_|*Pa* (*x*_*i*_)) represents the probability of occurrence of the child node *x*_*i*_ given the occurrence of its parent node. The joint probability denotes the expected likelihood of the observed variable *x*'s occurrence when multiple random variables satisfy their respective conditions, forming the foundation for prediction and inference.

### 2.5 Design of BN-HFACS

Based on the qualitative part of BN, we can combine it with HFACS. The human factors at each level of HFACS can be regarded as multiple variables in BN, and the relationship between the human factors at each level can be represented by directed edges in BN. The HFACS model's 4-level structure posits that all variables located in the higher level directly affect all variables located in the immediately lower level, which supports the construction of the BN.

(1) Designing the DAG: Our study is more concerned with the effect between the upper-level factors on the lower-level factors, so we assumed that sub-level factors in each HFACS level were independent and had no effect on each other. The organizational influence factor was considered the root node, while unsafe acts were treated as the leaf node. The model has been constructed following the progression of “organizational influences → unsafe supervision → preconditions for unsafe acts → unsafe acts.”

(2) Node state definition: In this study, all nodes were assigned two states- yes and no (representing the presence or absence of a factor in an accident). However, the error-type node had four states (skill, decision, perception, and none), and the violation-type node had three states (routine, exceptional, and none).

(3) Calculation of the CPT: A common approach for determining the CPT is parameter estimation based on a case database. In this study, we employed Netica 5.18 for calculations and analysis. Parameter learning was accomplished using a built-in case-learning algorithm. The case-learning database was constructed from the qualitative analysis results of 81 incident cases. This step enabled the calculation of the network node probability.

### 2.6 Sensitivity analysis

Sensitivity analysis is used to determine which upper-level factor contributes most significantly when a failure in an underlying factor occurs. This can be computed using the following equation (25), where *x*_*i*_ represents the upper-level factor, *x*_*j*_ denotes lower-level factor.


(2) $$\begin{array}{l} Sensitivity_{ij}=\left\{ \begin{array}{l} \frac{P(x_{j}=1|x_{i}=1)-P(x_{j}=1|x_{i}=0)\;}{P(x_{j}=1|x_{i}=0)},\;\Delta P\geq 0\\ \;0,\;\Delta P<0 \end{array}\right. \end{array}$$

*P*(*x*_*j*_ = 1|*x*_*i*_ = 1) represents the probability of occurrence of a lower-level factor *x*_*j*_ under the conditions of 100% existence (BN state = “yes”) of an upper-level factor. *P*(*x*_*j*_ = 1|*x*_*i*_ = 0) represents the probability of occurrence of a lower-level factor *x*_*j*_ under the conditions of 100% non-existence (BN state = “no”) of an upper-level factor. ΔP is *P*(*x*_*j*_ = 1|*x*_*i*_ = 1)−*P*(*x*_*j*_ = 1|*x*_*i*_ = 0 ).

## 3 Results

### 3.1 Qualitative analysis of incidents

**Figure 2:**
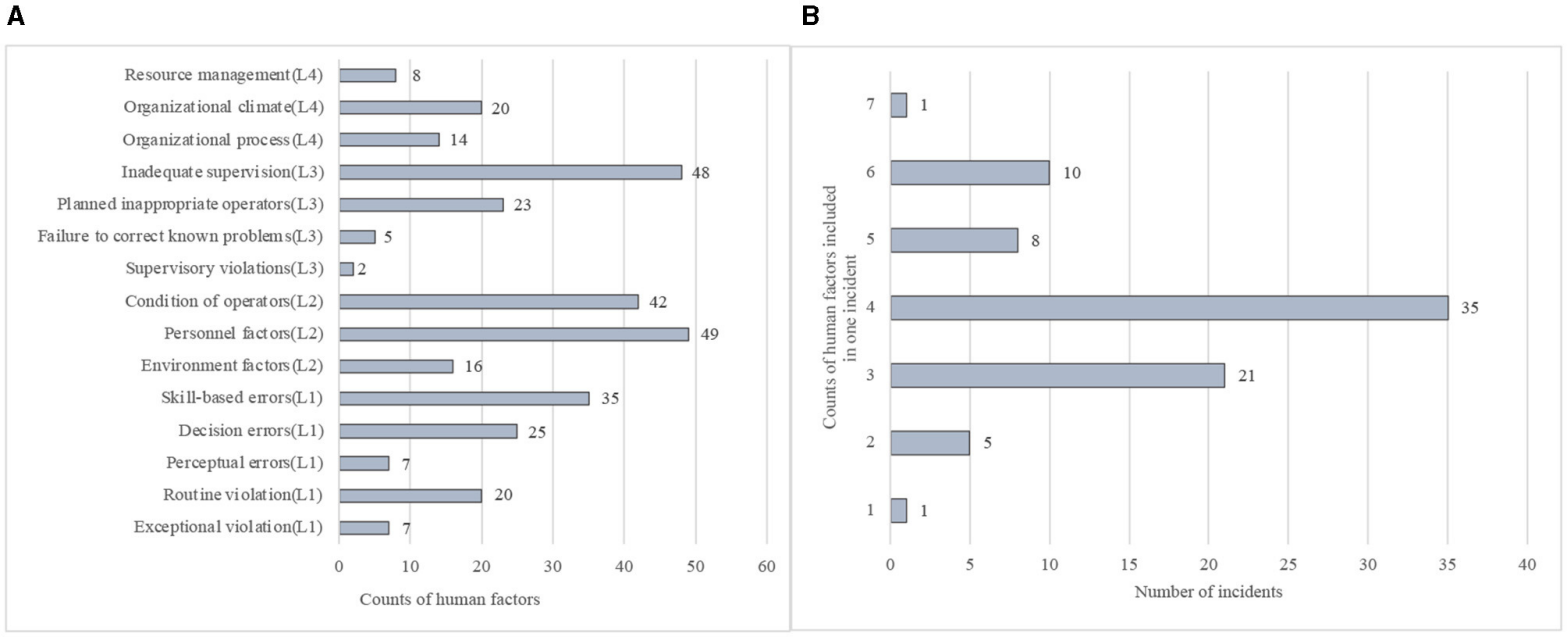
Results of qualitative analysis of incidents based on HFACS. **(A)** Frequency distribution of individual human factor subcategories involved in 650 incidents. **(B)** Frequency distribution of the number of human factors contained in a single incident.

Safety incidents can manifest at every stage of radiotherapy, from the initial patient assessment to the final delivery of treatment (Table 1). During the pre-treatment phase, errors in treatment planning constitute the largest proportion, especially “wrong data transfer or setting.” Incorrect patient setup, incorrect delivery, and delineation also demonstrated a higher likelihood of error, while simulation presented the lowest error incidence. Qualitative analysis utilizing the HFACS model revealed that among the four HFACS levels, unsafe acts made up 29.3% of reported human factors, preconditions for unsafe acts 33.3% unsafe supervision 24.3%, and organizational influences 13.1% (Figure 2A). In these 81 incident reports, 321 human factors were identified, with each report containing a minimum of one human factor and a maximum of six (Figure 2B).

**Table 1:** Statistics on the number of incidents in radiotherapy treatment phases and types of error.

**Phase**	**Error type**	**Description**	**Ratio**
Pre-treatment phase	Assessment of patient	ICD implantation is not considered	3/81
	Simulation	Wrong treatment field size	1/81
		Machine breakdown	1/81
	Delineation	Wrong definition	9/81
		Wrong CT image used	2/81
	Wrong registration	1/81
	Treatment planning	Wrong data transfer or setting	17/81
		Treatment history not considered	4/81
		Organ at risk dose out of limits	4/81
		Wrong structure copy	2/81
	Planning approval	The wrong treatment plan passed	4/81
Treatment phase	Patient exchange	Patient ID not verified	3/81
	Wrong patient set-up	Markers not correctly identified or wrong verification	15/81
	Wrong delivery	Repeated irradiation/missed irradiation	12/81
	No attention to the treatment room	Collision of gantry and couch	2/81
	Patient status was not observed	Patient has difficulty breathing	1/81

ICD, Implantable Cardioverter Defibrillators; CT, Computed Tomography.

### 3.2 BN-HFACS and priori probability of human factors

**Figure 3:**
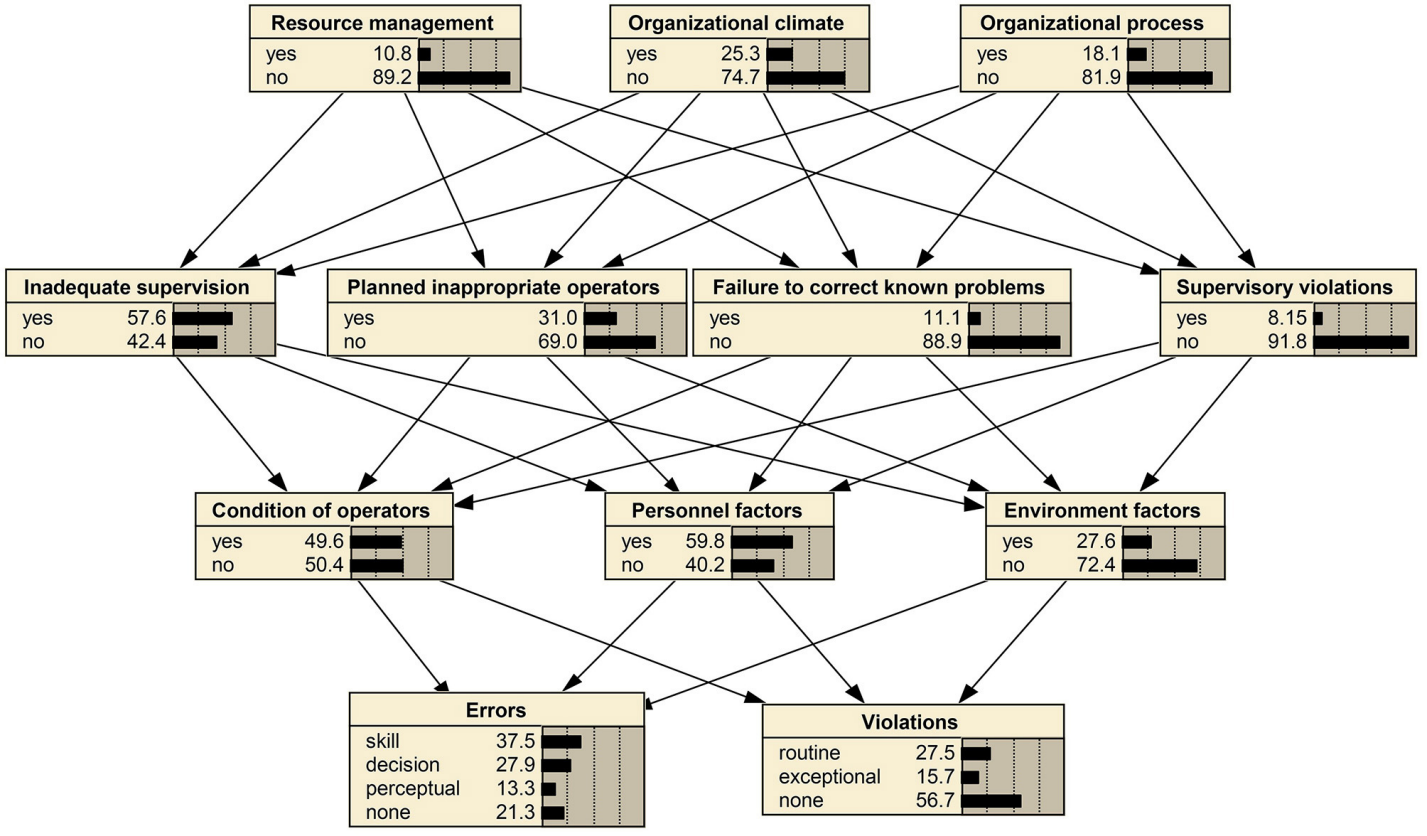
BN-HFACS network for analyzing human factors after the case-learning process. Based on this network, we can know the prior probability of different levels of human factors in radiotherapy safety incidents. For each factor, the greater the proportion of “yes,” the greater the a priori probability of that human factor.

The BN-HFACS was developed using Netica 5.18. Figure 3 illustrates the prior probability of each variable following the parameter learning process. At the level of unsafe acts, the highest prior probability (37.5%) was associated with skill-based errors. Interestingly, routine violations displayed a higher prior probability than exceptional violations. Condition of operators (49.6%) and personnel factors (59.8%) made up a large portion of preconditions for unsafe acts, whereas environmental factors demonstrated a smaller prior probability. Among factors at the unsafe supervision level, inadequate supervision had the highest prior probability (57.6%), followed by planned inappropriate operations. At the highest level, organizational climate had the most substantial prior probability (25.3%), largely reflecting issues such as safety culture awareness and the working atmosphere within the organization.

### 3.3 Sensitivity analysis of BN-HFACS

**Figure 4:**
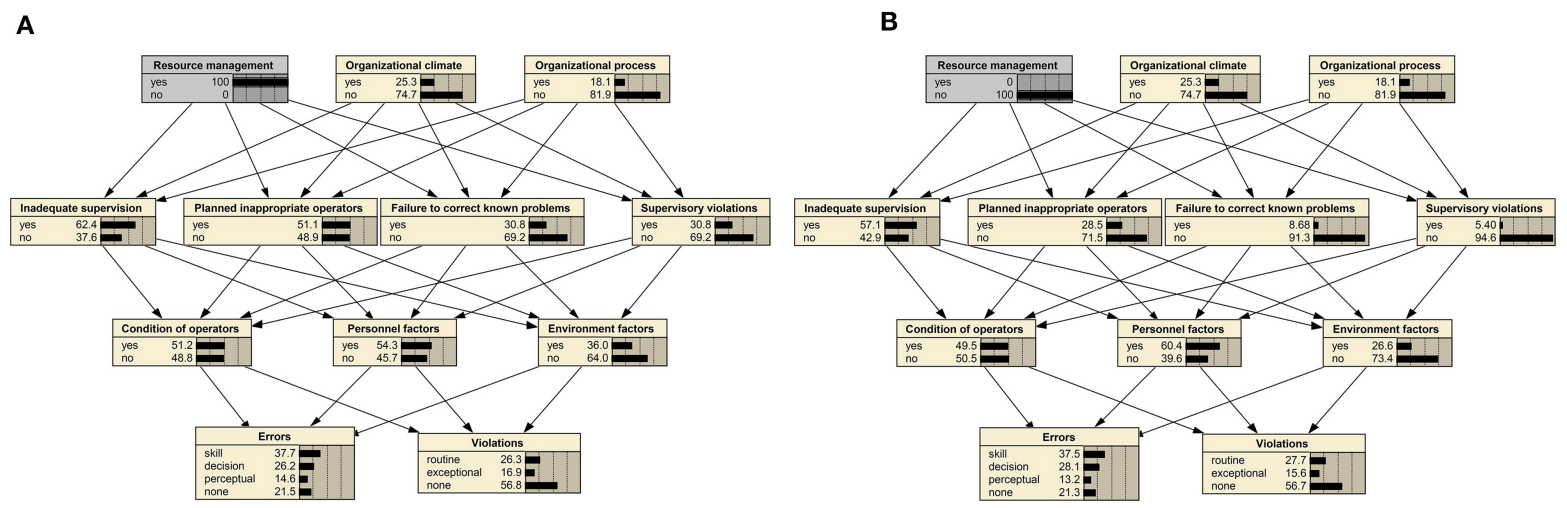
Change in probability of each node when there is a change in the state of resource management. **(A)** Assume a 100% probability of resource management issues being present in radiotherapy incidents. **(B)** Assume radiotherapy incidents are not at all relevant to resource management.

The sensitivity of a lower node to an upper node can be calculated using Equation (2) by altering the state of each node in the BN-HFACS. For instance, Figure 4 demonstrates the change in the state probability of lower nodes following the control of the state of the resource management node state. To illustrate the calculation process, sensitivity calculation for inadequate supervision is used as an example. As the state of resource management switched from “yes” to “no,” the probability of inadequate supervision shifted from 0.624 to 0.571. According to Equation (2), the sensitivity of inadequate supervision to resource management is (0.624–0.571)/0.571 = 0.09.

Sensitivity analysis demonstrated that different levels and human factors in the BN-HFACS had varying impacts on the individual types of errors and violations at Level 1 (Table 2). Among them, human factors at Level 2 exerted the most significant influence. Skill-based errors (SBE) were mainly influenced by conditions of operators (CO), with a sensitivity of 1.70. Decision errors (DE) and routine violations (RV) were primarily influenced by personnel factors (PF), with sensitivities of 0.74 and 0.46, respectively. Perceptual errors (PE) and exceptional violations (EV) were mainly influenced by environmental factors (EF), with sensitivities of 0.70 and 1.29, respectively.

**Table 2:** Sensitivity of nodes in BN-HFACS.

**Level**	**Factors**	**RM**	**OC**	**OP**	**IS**	**PIO**	**FCP**	**SV**	**CO**	**PF**	**EF**
Level 3	IS	0.09	3.87	0.00							
	PIO	0.79	1.81	0.00							
	FCP	2.55	0.00	0.98							
	SV	4.70	0.00	2.18							
Level 2	CO	0.03	0.33	0.02	0.02	0.14	0.10	0.05			
	PF	0.00	3.37	0.00	0.00	0.00	0.00	0.00			
	EF	0.35	1.62	0.17	0.00	0.00	0.97	0.95			
Level 1	SBE	0.01	0.01	0.37	0.01	0.05	0.03	0.00	1.70	0.08	0.00
	DE	0.00	0.00	0.00	0.00	0.00	0.00	0.00	0.00	0.74	0.00
	PE	0.11	0.05	0.05	0.02	0.10	0.15	0.27	0.10	0.00	0.70
	RV	0.00	0.00	0.00	0.00	0.00	0.00	0.00	0.25	0.46	0.00
	EV	0.08	0.06	0.04	0.00	0.00	0.21	0.21	0.00	0.00	1.29

RM, Resource management; OC, Organizational climate; OP, Organizational process; IS, Inadequate supervision; PIO, Planned inappropriate operators; FCP, Failure to correct known problems; SV, Supervisory violations; CO, Condition of operators; PF, Personnel factors; EF, Environment factors; SBE, skill-based errors; DE, decision errors; PE, perceptual errors; RV, routine errors; EV, exceptional errors. Columns indicate cause factors, and rows indicate result factors. The sensitivity index represents the degree of influence of the cause factors on the result factors, and the larger its value, the greater the influence of changes in the state of the cause factors on the result factors.

Human factors at Level 2 were prominently influenced by organizational climate (OC) at Level 4, particularly the heightened sensitivity of PF, which attained a value of 3.37. Among the three factors at Level 2, EF exhibited notable sensitivity not only to factors at Level 4 but also to the contributions of failure to correct known problems (FCP) and supervisory violations (SV) at Level 3. Conversely, CO and PF were nearly unaffected by Level 3.

Regarding human factors at Level 3, inadequate supervision (IS) and planned inappropriate operators (PIO) were mainly influenced by OC, with sensitivities of 3.87 and 1.81, respectively; FCP and SV were mostly affected by resource management (RM), with sensitivities of 2.55 and 4.70, respectively. Although the impact of organizational process (OP) on individual human factors at Level 3 may not be the most pronounced, it is noteworthy that PIO, FCP, and SV exhibited substantial sensitivity to OP, suggesting a non-negligible influence of OP on these factors.

**Figure 5:**
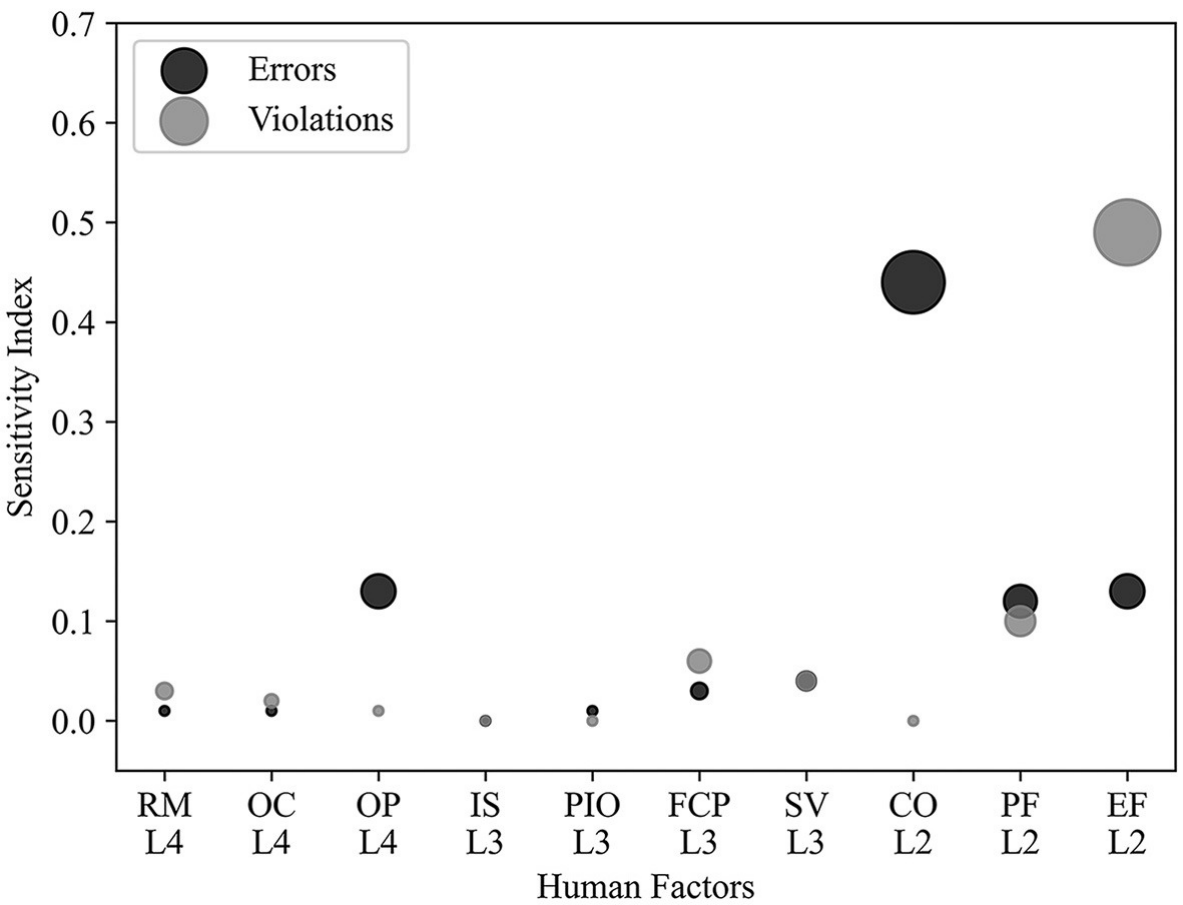
Sensitivity of overall errors and violations to upper-level factors, columns indicate cause factors, and rows indicate the degree of sensitivity. Size of the area of the circle reflects the degree of sensitivity.

To identify the most influential factors affecting overall errors and violations to prioritize control strategies, the absolute mean of each factor was computed across three states for overall errors (SBE, DE, and PE) and two states for overall violations (RV and EV). As depicted in Figure 5, it is evident that human factors at Level 2 still exert a more prominent influence. The errors and violations were primarily influenced by CO and EF, respectively. Additionally, we observed that the influence of OP on errors at the highest level is noteworthy and should not be overlooked.

## 4 Discussion

Radiotherapy-related incidents present a substantial risk to patient safety, making retrospective analysis of such incidents vital to improving safety measures (26, 27). In this study, we integrated HFACS with BN to create a potent quantitative tool capable of identifying key causal factors of unsafe acts in radiotherapy incidents. Employing the BN-HFACS model and sensitivity analysis, we identified specific relationships of different human factors within the RO-ILS publicly posted incidents, aiding the development of appropriate preventive measures.

Our qualitative analysis indicated that the most frequent errors across different clinical stages were in treatment planning, patient setup, and target delineation. During treatment planning, poor communication and incomplete documentation of policies and procedures can significantly impede organizational efficiency and contribute to planning errors (28–30). The most common issues within treatment planning were data transfer and parameter settings, indicating a need for standardization of process and written information to minimize verbal communication errors. Implementing systematic peer verification of information transfer is crucial to ensure accuracy in planning prior to approval.

To our knowledge, this study marks the first successful application of the BN-HFACS model in radiotherapy. While proactive risk assessment methods, such as failure modes and effects analysis, are widely used in radiotherapy (31, 32), they are often subjective. The BN-HFACS offers a systematic approach, helping to reduce the process's incompleteness when categorizing human factors due to limited expert knowledge or lack of information (33). BN allows for predictive or diagnostic reasoning (34), adding a significant advantage to this approach.

Although HFACS has been employed in the realm of radiotherapy in recent years, most studies have primarily used it to qualitatively analyze incidents, rarely examining the extent to which human factors at different levels contribute to incidents (5, 14, 16). Typically, during routine incident learning and analysis, the focus tends to be on the immediate causes leading to the incident and the individuals involved, while underlying factors are frequently overlooked (35). Our research effectively addressed this issue using the BN-HFACS model, facilitating the creation of targeted safety enhancements at the root level.

In the case-learning results of the BN-HFACS, skill-based errors, often resulting from the thoughtless execution of familiar tasks, had the highest prior probability of occurrence among various unsafe acts of workers. This finding aligns with previous studies in aviation, mining, and railways (6, 33, 36). Despite these industries being considerably different from radiation therapy, the human factors leading to safety incidents are interconnected and lessons can be learned from each other.

The preconditions for unsafe acts (Level 2) had the most significant influence on unsafe acts (Level 1). This correlation was confirmed for the first time in the field of radiotherapy. As unsafe acts are the most direct causes of incidents in radiotherapy, understanding their link with implicit factors is crucial for making substantial improvements. The condition of operators had the most substantial effect on skill-based errors, with factors such as inattention, mental fatigue, physical fatigue, and time pressure playing a role. One potential solution could be to rationalize the workload to alleviate these issues. This area certainly warrants our attention as we strive for long-term improvements. Our findings also indicate that personnel factors primarily influenced decision errors and habitual violations, while environmental factors predominantly affected perceptual errors and exceptional errors. Personnel factors generally stem from inadequate team communication and teamwork (15). In the context of radiotherapy, this is often attributable to doctors, physicists, and therapists operating in relatively independent roles, creating an environment where misinterpretations of intent and information bias can emerge, leading to errors (37). As for environmental factors, poor physical or technological environments, such as noise, insufficient lighting, and poorly designed equipment interfaces, can compromise staff attention and memory. Two incidents included in this study were caused by inadequate lighting in the treatment room, which led to incorrect positioning using the patient's tattoo as a reference line. Therefore, increasing the budget for maintenance or renovation of treatment rooms used for a prolonged period may enhance the treatment environment.

Simultaneously, we found that Level 2 factors were influenced by higher-level factors. Notably, the organizational climate has a significant impact, underlining the importance of cultivating a robust organizational safety culture to mitigate safety incidents. A positive climate includes staff awareness of radiotherapy's high-risk nature and a strong commitment to safety. It also indicates a blame-free work environment that encourages error reporting without fear of repercussions (38). To fortify an organization's safety culture, regular safety education is imperative.

Observing the upper-level factors and their impact on overall errors and violations, the importance of Level 2 factors remains evident. Additionally, organizational process exerts a notable influence on overall errors. Consequently, enhancing organizational policies and procedures is vital for reducing safety incidents.

Indeed, the unique structural configuration and operational arrangements of individual radiotherapy facilities inherently possess distinct characteristics, which could potentially result in the varying occurrence of safety incidents and contributing factors. However, this paper presents a methodological framework for incident analysis, empowering each facility to conduct a thorough evaluation of both qualitative and quantitative facets, and the process of analyzing RO-ILS incidents can also be used as a reference for independent agencies using the BN-HFACS model. By doing so, it enables the identification of particularly vulnerable aspects within the clinical radiotherapy process and the discernment of the most significant contributing factors. Therefore, this approach supports the development of focused preventive measures in each treatment center, customized to effectively tackle these identified weaknesses.

However, this study does have some limitations. Firstly, the sample size is relatively small. While a sample of 40 cases was deemed sufficient for accurate BN parameter learning (25), a larger sample size could improve the generalizability of the findings. Owing to the sample size limitations, this study did not specifically address the severity of the included incidents. Further segmentation and analysis might potentially yield more unique insights. Additionally, safety incidents have been reported by a range of institutions, and the completeness of incident reporting cannot be fully assured. Moreover, retrospective analysis can present challenges in determining the origins of events and HFACS factors. Nevertheless, these limitations are acceptable considering the inherent nature of large international incident learning systems (15). Last but not the least, our analysis only targeted incidents reported in the RO-ILS, which may not represent the actual errors occurring worldwide. In order to enhance the generalizability of our findings, larger and more database in multiple regions is required in the future.

## 5 Conclusion

In conclusion, the analysis of 81 radiotherapy incidents utilizing the BN-HFACS model brought to light significant associations between the implicit factors and unsafe acts in radiotherapy incidents. Sensitivity analysis demonstrated that the preconditions for unsafe acts (Level 2) had the most considerable impact on unsafe acts (Level 1). Organizational climate and process surfaced as the profound potential factors contributing to final incidents. To enhance radiotherapy safety, it is crucial to implement measures that focus on four-levels human factors of HFACS, thereby reducing the likelihood of unsafe acts originating from their root causes.

## Data availability statement

The original contributions presented in the study are included in the article/supplementary material, further inquiries can be directed to the corresponding author.

## Author contributions

HH: Writing – original draft, Data curation, Formal analysis. XP: Writing – original draft, Data curation, Formal analysis. DL: Methodology, Writing – original draft. WW: Methodology, Writing – original draft. JL: Methodology, Writing – original draft. QW: Data curation, Formal analysis, Writing – original draft. QX: Data curation, Formal analysis, Writing – original draft. GL: Supervision, Validation, Writing – review & editing, Funding acquisition. SB: Supervision, Validation, Writing – review & editing, Funding acquisition.
